# Impact of the COVID-19 pandemic on training conditions and education in oncologic disciplines: a survey-based analysis

**DOI:** 10.1007/s00066-023-02121-6

**Published:** 2023-08-04

**Authors:** Maike Trommer, Anne Adams, Carolin Bürkle, Stefanie Brunner, Andrea Grandoch, Alexandra Geffroy, Cindy Franklin, Asmae Gassa, Anna-Katharina Meißner, Sibylle Mellinghoff, Shachi Jenny Sharma, Silvia Schrittenlocher, Luisa Witte, Simone Marnitz

**Affiliations:** 1 Klinik und Poliklinik für Radioonkologie, Cyberknife- und Strahlentherapie, Medizinische Fakultät und Uniklinik Köln, Universität zu Köln, Kerpener Str. 62, 50937 Cologne, Germany; 2 Center for Integrated Oncology (CIO), Medizinische Fakultät und Uniklinik Köln, Universität zu Köln, Cologne, Germany; 3 Center for Molecular Medicine Cologne, Medizinische Fakultät und Uniklinik Köln, Universität zu Köln, Cologne, Germany; 4 Institut für Medizinische Statistik und Bioinformatik, Medizinische Fakultät, Universität zu Köln, Cologne, Germany; 5 Medizinische Klinik und Poliklinik III, Ludwig-Maximilians-Universität (LMU), Munich, Germany; 6 Klinik und Poliklinik für Allgemein‑, Viszeral‑, Tumor- und Transplantationschirurgie, Medizinische Fakultät und Uniklinik Köln, Universität zu Köln, Cologne, Germany; 7 Klinik für Poliklinik für Mund-Kiefer- und Plastische Gesichtschirurgie, Medizinische Fakultät und Uniklinik Köln, Universität zu Köln, Cologne, Germany; 8 Klinik für Frauenheilkunde, Universitätsklinikum Freiburg im Breisgau, Medizinische Fakultät, Universität Freiburg, Freiburg im Breisgau, Germany; 9 Klinik und Poliklinik für Dermatologie und Venerologie, Medizinische Fakultät und Uniklinik Köln, Universität zu Köln, Cologne, Germany; 10 Klinik und Poliklinik für Herzchirurgie, herzchirurgische Intensivmedizin und Thoraxchirurgie, Herzzentrum an der Universität zu Köln, Medizinische Fakultät und Uniklinik Köln, Universität zu Köln, Cologne, Germany; 11 Zentrum für Neurochirurgie, Klinik für Allgemeine Neurochirurgie, Medizinische Fakultät und Uniklinik Köln, Universität zu Köln, Cologne, Germany; 12 Medizinische Klinik I, Medizinische Fakultät und Uniklinik Köln, Universität zu Köln, Cologne, Germany; 13 Klinik für Hals‑, Nasen‑, Ohrenheilkunde, Kopf und Halschirurgie, Medizinische Fakultät und Uniklinik Köln, Universität zu Köln, Cologne, Germany; 14 Zentrum für Augenheilkunde, Medizinische Fakultät und Uniklinik Köln, Universität zu Köln, Cologne, Germany; 15 Urologische Klinik, Bundeswehrkrankenhaus Ulm, Ulm, Germany

**Keywords:** Corona pandemic, Cancer health services research, Further education, Oncology training, Educational development

## Abstract

**Purpose:**

The COVID-19 pandemic has led to changes in global health care. Medical societies had to update guidelines and enhance new services such as video consultations. Cancer treatment had to be modified. The aim of this study is to ensure optimal care for cancer patients with the help of high-quality training even in times of crisis. We therefore conducted a nationwide survey of physicians in training in oncological disciplines during the pandemic to assess the impact on their education.

**Methods:**

The survey was sent to tumour centres, hospitals, specialist societies, and working and junior research groups and distributed via newsletters and homepages. Interim results and a call for participation were published as a poster (DEGRO) [26] and in the German Cancer Society (DKG) journal *FORUM* [42]. The survey contained 53 questions on conditions of education and training and on clinical and scientific work. Statistics were carried out with LimeSurvey and SPSS (IBM Corp., Armonk, NY, USA).

**Results:**

Between February and November 2022, 450 participants answered the survey, with radio-oncologists being the largest group (28%). Most colleagues (63%) had access to digital training methods. Virtual sessions were rated as a good alternative, especially as multidisciplinary meetings (54%) as well as in-house and external training programs (48%, 47%). The time spent by training supervisors on education was rated as less than before the pandemic by 57%. Half of all participants perceived communication (54%), motivation (44%) and atmosphere (50%) in the team as bad. The participants felt strongly burdened by extra work (55%) and by a changed team atmosphere (49%). One third felt a change in the quality of training during the pandemic and rated it as negative (35%). According to 37% of the participants, this had little influence on their own quality of work. Additional subgroup analyses revealed significant differences in gender, specialty and education level.

**Conclusion:**

In order to improve oncology training in times of crisis, access to digital training options and meetings should be ensured. Participants wish for regular team meetings in person to enable good team spirit, compensation for overtime work and sufficient time for training supervisors for discussion and feedback.

**Supplementary Information:**

The online version of this article (10.1007/s00066-023-02121-6) contains supplementary material, which is available to authorized users.

## Introduction and purpose

The coronavirus disease of 2019 (COVID-19) pandemic led to drastic changes in healthcare worldwide [1]. In order to ensure the treatment of patients suffering from COVID-19, prioritisation categories for patient care had to be defined, which ensured adequate treatment of other diseases despite reduced capacities [2]. Many medical societies have therefore published updates to their guidelines [3–9], which are constantly being refined in line with the changing pandemic situation [10, 11]. To save resources in intensive care units and shorten hospital stays, the treatment of oncological patients was also adapted, for example by limiting inpatient treatments, modifying treatments such as hypofractionation of radiation or reduction of chemotherapeutic interventions, changes in palliative care and screening programmes [12–16]. Possible radiation oncology treatment concepts for times of crisis were provided with evidence-based pragmatic fractionation schemes to accommodate limited staff capacity, while cautioning against compromising oncological outcomes. It was also recommended to consider equally effective non-invasive radiotherapy and radiochemotherapy as alternatives to surgical treatments to save resources for surgical and anaesthesiologic capacity [17]. Medical care had to be redesigned. New services were implemented and existing concepts expanded, such as telephone and video consultations or virtual meetings [18]. Quarantine periods, distance rules, visiting bans and “social distancing” also affected (psycho-)oncological and social care [19–21].

Employees in oncology care are not only active administratively and clinically, but must also ensure outstanding research and education in their specialist disciplines [22].

In the pre-COVID era, there was already a rising trend in the field of education towards digital teaching and modernising educational practices. However, this was not fully implemented nationwide. Privately, the use of social media and apps was common in young adults [23] but digital media were not commonly part of education at German universities before the pandemic [24]. However, the use of digital technology in various formats had begun to emerge [25]. The pandemic acted as a catalyst, accelerating the adoption of digital teaching methods and innovative approaches to teaching and learning [26, 27].

The scope and availability of oncology education and training programmes changed noticeably during the pandemic. Congresses and events were held in alternative (online) formats where possible. The additional adoption of alternative educational formats such as digital options (online teaching) may have had an impact on training quality in oncological disciplines and thus directly and indirectly on oncological patient care.

COVID-associated changes may have endangered qualitative and structured education during residency training. Given the significant potential impact, we conducted a nationwide survey among physicians undergoing training in oncological disciplines during the COVID-19 pandemic. The objective of this study was to comprehensively document and structure the impact of the pandemic across various domains, along with the associated challenges. We addressed questions regarding the alterations in training conditions, the most influential factors affecting training, and the potential impact on the quality of work and patient care. Furthermore, we provide subgroup analyses based on gender, specialty, and education level. Our ultimate aim is to promote positive aspects and enhance the medical care provided to cancer patients by ensuring high-quality training in times of crisis and beyond.

## Materials and methods

The project was developed within the framework of a funded programme for female physicians at the University Hospital Cologne (MhÄK). The survey was created in January 2022 using the platform LimeSurvey University of Cologne v. 3.27.26 and contained 53 questions distributed over 9 pages on various areas of education and training as well as on everyday clinical and scientific work in oncological care during the pandemic. The individual questions covered the areas of person-related questions (age, gender, workplace and level of training), COVID-specific questions on the scope of work and specialisation focus, meetings and events within the team and in training, personal feelings and everyday work. Participation was anonymous and voluntary, and could be interrupted or cancelled at any time. A pre-test was carried out in a group of 10 colleagues working in oncology in order to check the comprehensibility, accessibility and technical compatibility as well as any limitations, and to make appropriate corrections.

The survey and a reminder were sent to a total of 31 specialist societies as well as working and junior research groups during the first semester of 2022, distributed via their newsletters and published on the corresponding homepages. The participating groups and their German abbreviations are listed in alphabetical order in Table 1.

**Table 1:** Participating groups and their German abbreviation in alphabetical order

German abbreviation	Groups involved
ABO	Working Group Imaging in Oncology
ACO	Association Surgical Oncology
ADO	Working Group Dermatological Oncology
AGO	Working Group Gynaecological Oncology
AHMO	Working Group Otorhinolaryngology, Oral and Maxillofacial Oncology
AIO	Association of Internal Oncology
AOT	Association of Oncological Thoracic Surgery
APM	Working Group Palliative Medicine
APO	Working Group Paediatric Oncology
ARO	Working Group Radiological Oncology
AUO	Working Group Urological Oncology
CAJC	Young Surgery Working Group
CAO	Surgical Association of Oncology
DEGRO	German Society for Radiooncology
DGHNO	German Society of Otorhinolaryngology
DGHO	German Society for Haematology and Medical Oncology
DGNC	German Society of Neurosurgery
DGP	German Society for Pathology
DGSP	German Society for Sports Medicine
DKG	German Cancer Society
GeSRU	German Society of Residents in Urology
IAG-FIO	Interdisciplinary Working Group Women in Oncology of the DKG
NOA	Neurooncology Working Group
POA	Pneumologica l Oncology Association
PSO	Working Group for Psychooncology
YOU	Young Oncologists United
YTO	Young Thoracic Oncologists
	Chirurginnen e. V.
	Young DGHO
	Young DGHNO
	Young Surgeons

The survey was also sent to tumour centres, hospital and practice groups and their directors, and asked to be distributed among trainees. In order to achieve the greatest possible reach, flyers were distributed at training events at congresses and interim results and a call for participation were presented as a poster (DEGRO) [28]. In addition, there was a call for participation in the DKG journal *FORUM* (vol. 37, iss. 3, 06/2022) [42].

Statistics and figures were produced using LimeSurvey v. 3.27.26, SPSS v. 28 (IBM Corp, Armonk, NY, USA) and Microsoft Excel 2021 (Redmond, WA, USA). Descriptive statistics were calculated for the demographic data. Participant characteristics and subgroups were compared by the Kruskal–Wallis test for continuous variables and Pearson’s chi-square test for categorical variables where appropriate. In any case, *p*-values < 0.05 were considered significant. For subgroup analyses, we included gender (male and female), specialty (surgical and non-surgical disciplines) and education level (university degree and higher degree [Dr. med. or Priv.-Doz., PD]). Surgical disciplines included general/visceral/thoracic surgery, gynaecology, neurosurgery, ear/nose/throat, dermatology, maxillofacial surgery, urology, ophthalmology and orthopaedics.

Not all questions had to be answered, so the number of complete answers per questionnaire varied.

The project received a positive vote from the Ethics Committee of the Medical Faculty of the University of Cologne (reference 22-1367_1).

## Results

### Characteristics

Of a total of 497 participants, 450 processed the survey and 291 completed all questions. 275 (62%) female colleagues and 168 (38%) male colleagues participated. The majority of respondents were female (62%) and under 36 years old (*n* = 256, 58%; ≤ 30 years. *n* = 98; 31–35 years. *n* = 158). The highest level of education ranges from state examination (*n* = 113, 26%) to doctorate (*n* = 292, 66%) to habilitation/venia legendi (Priv.-Doz., PD; *n* = 37, 8%). Whereas 242 (55%) participants were residents, 169 (38%) were specialists and consultants (Table 2).

**Table 2:** Characteristics of the participants

Variable		Response *n* (%)
Entire cohort		450 (100%)
Age group	< 30 years	98 (22%)
	31–35 years	158 (36%)
	36–40 years	78 (18%)
	> 40 years	19 (25%)
Gender	Male	168 (38%)
	Female	275 (62%)
	Diverse	0 (0%)
Educational qualification	University degree	113 (26%)
	Medical doctorate (Dr. med.)	292 (66%)
	Habilitation/venia legendi (PD)	37 (8%)
Same department before pandemic	Yes	353 (80%)
	No	90 (20%)
Specialty	Radiation oncology	118 (26%)
	Medical oncology	108 (24%)
	General/visceral/thoracic surgery	31 (7%)
	Gynaecology	29 (6%)
	Neurosurgery	27 (6%)
	Ear/nose/throat	26 (6%)
	Dermatology	22 (5%)
	Radiology and nuclear medicine	15 (3%)
	Maxillofacial surgery	13 (3%)
	Urology	10 (2%)
	Paediatrics	8 (2%)
	Pathology	5 (1%)
	Ophthalmology	4 (1%)
	Palliative care	4 (1%)
	Neurology	3 (1%)
	Orthopaedics	3 (1%)
	Other	24 (5%)

The most frequently represented disciplines were radiation oncology (*n* = 118, 26%), medical oncology (*n* = 108, 24%), surgical disciplines (*n* = 30, 7%) and gynaecology (*n* = 29, 7%). The information given in the text field “other” was also considered in this calculation (Table 2), e.g., “*Strahlentherapie*” was added to “radiation oncology”.

A total of 381 (85%) of the respondents work in a university or primary care hospital, and 66 (15%) work in a medical practice or a peripheral hospital. The majority (*n* = 353, 80%) had already worked at their current workplace before the pandemic.

### Scope and extent of work and specialisation focus

Overall, 45% (*n* = 183) of the participants stated that they had more clinical workload during compared to before the pandemic. 58% (*n* = 239) of the participants answered that they had additional pandemic-specific tasks (e.g., implementing hygiene concepts, conducting COVID swabs, special visit authorisations) and 55% (*n* = 226) a lack of resources (e.g., lack of staff); 14% (*n* = 56) cited their personal environment (e.g., lack of childcare) as the reason for a change in workload. 28% (*n* = 114) of the participants stated that they had no capacity for scientific work due to the clinical workload.

For 51% (*n* = 209), the scope of work remained unchanged during the pandemic. 147 (36%) colleagues were temporarily stationed elsewhere. This had a negative influence on training in 109 (27%) cases, 28% indicated no influence, 1% a positive influence.

### Meetings within the team and multidisciplinary meetings (tumour boards)

Before the pandemic, team meetings were usually (90%) held face to face. During the pandemic, 57 (15%) colleagues had no access to team meetings at all, 131 (34%) virtually and 86 (22%) continued to have face-to-face meetings. In 25% of the cases, both formats were offered. Overall, 37% (*n* = 142) had less or no access to team meetings. Team meetings were held with a reduced number of people in 53% (*n* = 207). The change in the extent and group size of team meetings had a subjectively negative effect on training in 32% (*n* = 124) of the cases, 37% indicated no influence, 6% a positive influence.

Oncological multidisciplinary case conferences, so-called tumour boards, were mostly (78%) held in face-to-face presence before the pandemic. During the pandemic, 55 (15%) colleagues had no access to tumour boards at all, while 174 (47%) were able to participate virtually, 56 (15%) in presence and 83 (22%) in both formats. Overall, 33% (*n* = 121) had less frequent or no access at all. Tumour boards were held in reduced numbers for 52% (*n* = 193) of participants. The change in extent and group size had a subjectively negative effect on training in 25% (*n* = 92) of the cases, 38% said it had no effect and 9% said it had a positive effect.

### In-house and external training programmes and events

**Fig. 1:**
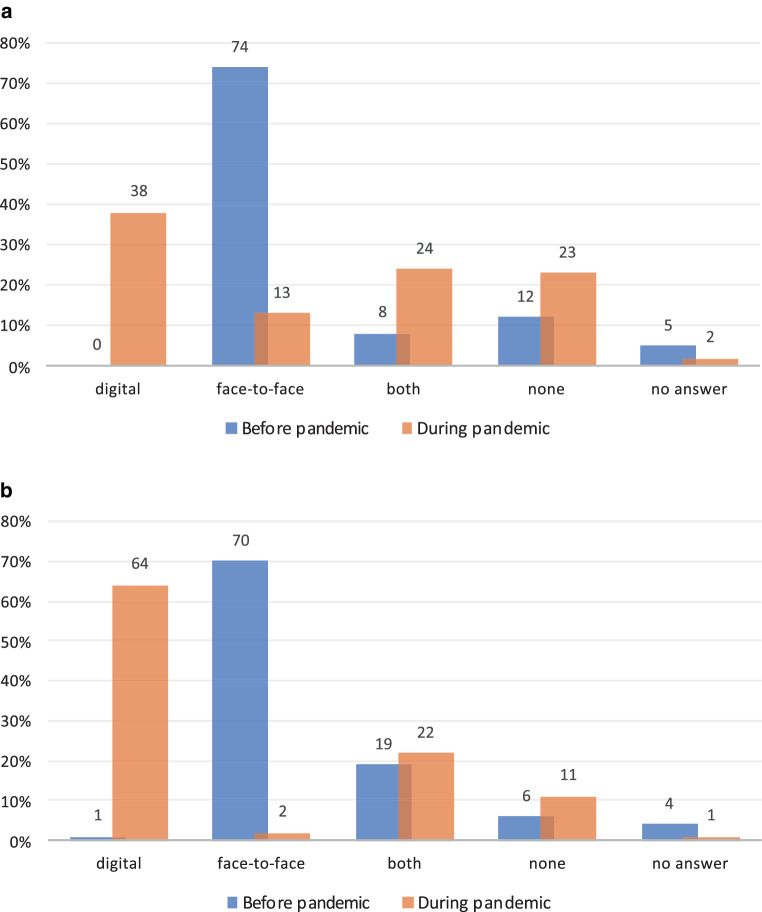
Answers to access to in-house (**a**) and external (**b**) training and professional development events before (*blue*) and during (*orange*) the pandemic

Before the pandemic, 74% (*n* = 249) of colleagues had access to in-house training or professional development events such as journal club or weekly in-person training, while 12% (*n* = 41) had no access. During the pandemic, 38% (*n* = 126) of participants were able to attend in-house events virtually, 13% (*n* = 45) still had face-to-face access and 24% (*n* = 81) had both. Overall, 23% (*n* = 77) of participants had no access to in-house training or professional development events (Fig. 1a).

The majority (62%, *n* = 185) stated that planned in-house events were cancelled without substitution. Alternative formats of in-house events were cited by 193 (65%) colleagues as substitutes. This improved availability in 75 (22%) cases and worsened it in 95 (28%) cases. Professional exchange deteriorated in most cases (*n* = 198, 59%), personal contacts and networking also deteriorated in 242 (72%) cases.

Before the pandemic, 223 (70%) colleagues had access to external training or professional development events such as congresses, specialist courses, etc. in presence, 6% (*n* = 18) had no access. During the pandemic, 64% (*n* = 204) of participants were able to attend external events virtually, 1% (*n* = 5) were able to attend face-to-face events and 22% (*n* = 71) were able to attend both formats. 11% (*n* = 36) of the participants had no access at all to external training or professional development events (Fig. 1b).

Planned external events had been cancelled without substitution in 69% (*n* = 203) of the responses. Alternative formats (digital) of external events were given as substitutes by 282 (95%) colleagues. Availability was better in 132 (42%) cases and worse in 83 (26%) cases. Costs were reported as better in 173 (54%) cases and unchanged in 78 (25%) cases. Professional exchange deteriorated in most cases (*n* = 241, 76%), personal contacts and networking also deteriorated in 257 (81%) cases.

Access to clinical training options in direct contact with patients was limited or very limited in 184 (62%) cases during the pandemic. Few alternatives were provided as a substitute during the pandemic: live online teaching (*n* = 59, 20%) or e‑learning platform (*n* = 43, 14%). In 54% (*n* = 162) of the cases there was no substitute.

### Alternative formats (digital) for training

**Fig. 2:**
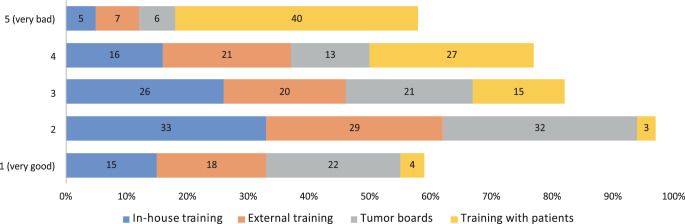
Answers to the question of whether digital formats were a good or bad alternative to face-to-face events (in percent): in-house (*blue*) and external (*orange*) training events; tumour boards (*grey*) and training options with contact to patients (*yellow*)

The participants rated the following digital events according to a scale from very poor to very good alternative to face to face. Good or very good ratings were given to tumour boards in 161 (54%) cases, in-house professional development events in 142 (48%) cases, external professional development events in 139 (47%) cases and team meetings with training character in 123 (41%) cases. Training options with contact to patients were rated as poor in the majority of cases (*n* = 197, 66%; Fig. 2).

For alternative formats, 63% (*n* = 187) said they had the necessary technical and physical requirements, 9% (*n* = 26) were overchallenged with this. 36% (*n* = 106) reported frequent technical complications that make this type of training method difficult. A total of 33% (*n* = 99) of the participants stated that their training suffered due to alternative digital formats. A total of 41% (*n* = 123) stated that it was easy for them to continue their education in this way and approximately the same number (37%; *n* = 110 and *n* = 109) stated that they enjoyed or did not enjoy continuing their training with alternative formats.

### Communication and team

**Fig. 3:**
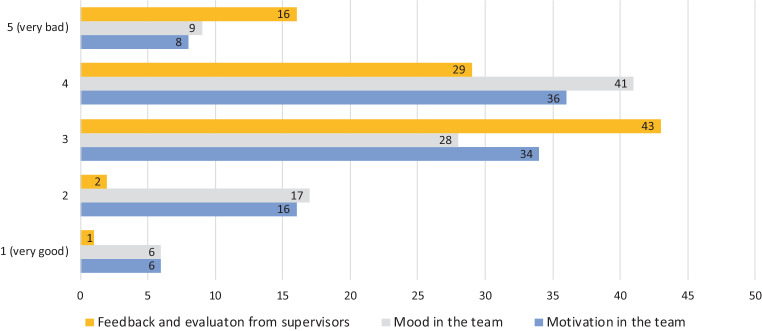
Feedback and evaluation of training status from supervisors during the pandemic compared to before the pandemic (*yellow*); mood (*grey*) and motivation (*blue*) in the team during the pandemic compared to before the pandemic

In half of the cases (49%, *n* = 145), the dialogue with superiors, training supervisors or experienced colleagues during the pandemic was less frequent than before the pandemic. Feedback on the status of training was stated as bad to very bad in 45% (*n* = 133; Fig. 3).

Personal appraisals were held less frequently in 41% (*n* = 123) of cases. 142 (57%) colleagues rated the time that training supervisors could take for their training as less than before the pandemic. 54% (*n* = 151) of the participants stated a poorer communication in the team and 42% (*n* = 118) a poorer cohesion in the team than before the pandemic. Overall, 122 (44%) colleagues perceived the motivation in the team and 138 (50%) colleagues perceived the atmosphere in the team to be bad (Fig. 3).

### Personal well-being, pressures and everyday work

The question about their own motivation was answered by 43% (*n* = 121) of the participants with good and 19% (*n* = 53) with poor. The quality of their own work tended to be assessed as good by 64% (*n* = 174).

Their own satisfaction at work was equally better or worse (35%). 101 (37%) colleagues stated that their personal well-being was good, 79 (29%) answered that it was poor. Overall, 113 (41%) participants were able to maintain their work satisfaction, 69 (25%) indicated a significant deterioration.

**Fig. 4:**
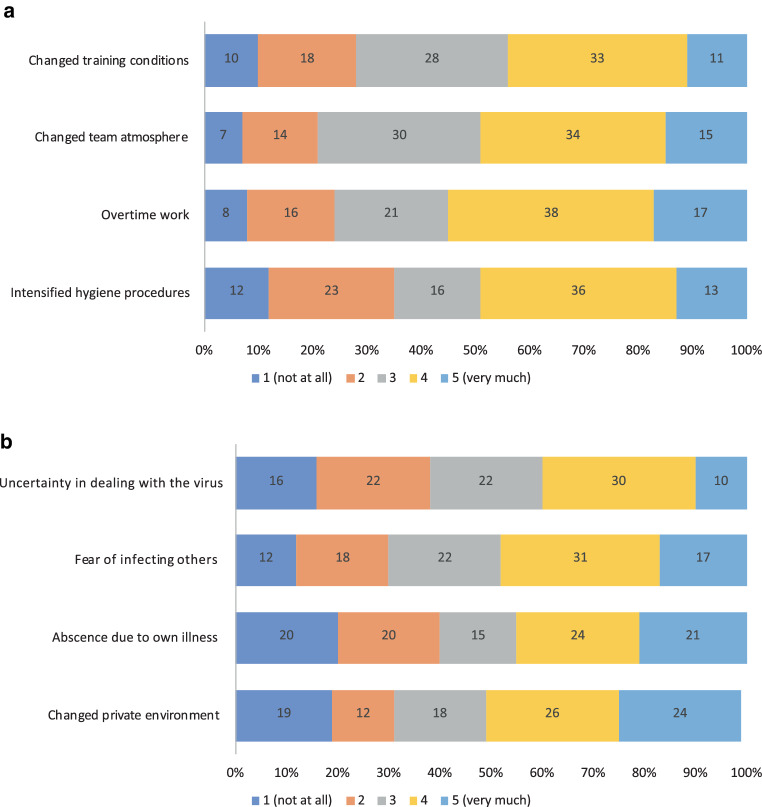
The most common reasons for work-related (**a**) and personal (**b**) pressures during the pandemic

In terms of their everyday work, the participants felt, in decreasing order, pressured by extra work (55%; *n* = 152), by intensified hygiene procedures such as wearing a mask (49%; *n* = 139), by a changed atmosphere in the team (49%; *n* = 136), by changed training conditions (43%; *n* = 119), by changed clinical/practice procedures (38%; *n* = 106), by fewer opportunities to consult with experienced colleagues (36%; *n* = 97) and due excessive demands (27%; *n* = 76; Fig. 4a).

A change in the private environment, such as the lack of childcare facilities (50%, *n* = 126), the fear of infecting others (48%, *n* = 136), the absence due to one’s own illness (45%, *n* = 120), the initial uncertainty in dealing with the virus (40%, *n* = 110) and the fear of getting infected oneself (26%, *n* = 74) also led to strong personal pressures (Fig. 4b).

### Factors influencing the quality of training conditions and own work

The discontinuation or reduction of face-to-face events was rated by half of the respondents (144, 50%) as a strong influencing factor for an impairment of training conditions during the pandemic. In particular, discontinuation or reduction of external training (47%), in-house training (41%), team meetings (36%) and tumour boards (25%) was rated as detrimental.

**Fig. 5:**
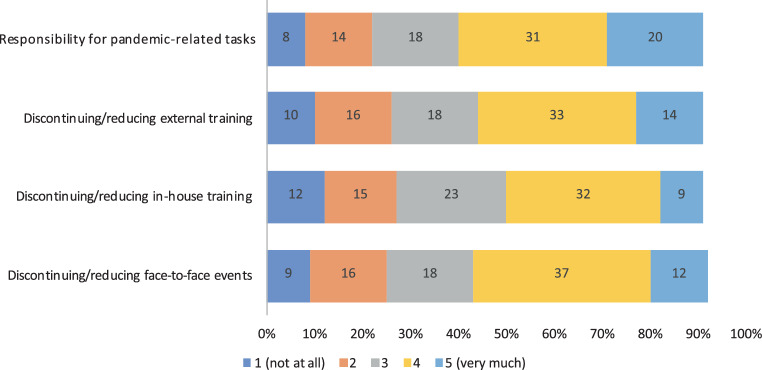
The biggest negative influencing factors on training conditions during the pandemic

With regard to everyday work, 149 (51%) colleagues rated the responsibility for pandemic-related tasks (swabs, visiting regulations, hygiene procedures, more documentation work) as a strong factor for an impairment of training conditions during the pandemic; 73 (25%) the reduction of operating times, interventions or elective treatments; 71 (24%) reduced time spent in their specialty area; and 70 (24%) their own absence from work due to illness, quarantine, etc. (Fig. 5).

**Fig. 6:**
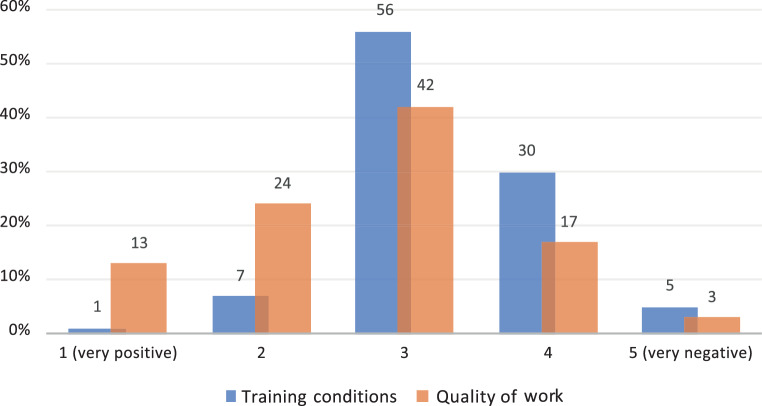
Impact of the coronavirus pandemic on training conditions (*blue*) and quality of work (*orange*)

Overall, 33% (*n* = 96) of the survey participants stated that the quality of training changed strongly during the coronavirus pandemic. According to 37% (*n* = 108) of the participants, the pandemic-related changes had little influence on their own quality of work. The quality of training during the pandemic was rated as good by 34% (*n* = 98) and poor by 28% (*n* = 80). The coronavirus pandemic affected personal training negatively for 102 (35%) colleagues and positively for 25 (8%) (Fig. 6).

### Subgroup analysis

For subgroup analyses, we included gender (male and female), specialty (surgical and non-surgical disciplines) and education level (university degree and higher degree such as Dr. med. or Priv.-Doz., PD; Table 3). We could not include age in the analysis due to a significantly different distribution of gender and education level within the age groups (Supplementary Table 1): female gender was found significantly more often in the age group ≤ 35, male gender in the age group > 40 (*p* = 0.007). University degree was significantly more represented within the age group ≤ 35 and higher degrees in the age group > 40 (*p* = 0.001; Supplementary Table 1). We have only displayed significant differences in the responses within the subgroups in Table 3. More results can be seen in Supplementary Table 2.

**Table 3:** Significant differences in answers of subgroups

Question	Group	Response, *n* (%)	*p*-value
**Working conditions during the pandemic**			
*No change of work scope or extent*			
	University degree	23 (23%)	0.027
	Higher degree (MD, PD)	109 (35%)	
*More clinical work*			
	Surgical	49 (32%)	< 0.001
	Non-surgical	134 (52%)	
*Transferred to different field (not original field of education)*			
	Surgical	42 (28%)	0.006
	Non-surgical	105 (41%)	
*Less access to childcare*			
	Male	14 (9%)	0.032
	Female	42 (17%)	
	Surgical	13 (9%)	0.018
	Non-surgical	43 (17%)	
**In-house training**			
*No access during pandemic*			
	Male	20 (16%)	0.019
	Female	57 (27%)	
*No alternatives to replace face-to-face education content*			
	Surgical	78 (68%)	< 0.001
	Non-surgical	84 (46%)	
**Personal elaboration of cancelled education content**			
	Male	49 (44%)	0.018
	Female	52 (28%)	
**Less feedback from supervisors**			
	Male	55 (50%)	0.009
	Female	68 (37%)	
**Personally rated quality of own work: good and very good**			
	Male	58 (57%)	0.022
	Female	116 (67%)	
**Pressure due to …**			
*Excessive demands*			
	Male	20 (20%)	0.046
	Female	56 (31%)	
	Surgical	19 (17%)	0.043
	Non-surgical	57 (33%)	
*Less communication with experienced colleagues*			
	University degree	35 (56%)	< 0.001
	Higher degree (MD, PD)	62 (30%)	
*Fear to infect others*			
	Male	42 (41%)	0.001
	Female	94 (52%)	
**Biggest negative influencing factors on training conditions**			
*Discontinued/reduced face-to-face events*			
	Male	44 (40%)	0.035
	Female	60 (33%)	
	Surgical	33 (30%)	0.050
	Non-surgical	71 (39%)	
*Discontinued/reduced in-house training*			
	Male	39 (36%)	0.031
	Female	79 (43%)	
	Surgical	36 (33%)	0.003
	Non-surgical	82 (45%)	
*Discontinued/reduced elective interventions*			
	Surgical	51 (46%)	< 0.001
	Non-surgical	22 (12%)	
*Being transferred to different field of work*			
	Surgical	36 (33%)	0.001
	Non-surgical	35 (19%)	

Regarding female and male participants, there were significantly different answers to the question of whether work conditions changed during the pandemic because of less access to childcare facilities (female *n* = 42, 41%; male *n* = 14, 9%; *p* = 0.032), female participants had significantly less access to in-house training during the pandemic (*p* = 0.019), could significantly less often elaborate cancelled education content on their own (*p* = 0.018), got significantly more feedback from their supervisors (*p* = 0.009) and rated their own quality of work significantly more often as good and very good (*p* = 0.022). Women felt strongly burdened significantly more often by excessive demands at work (*p* = 0.046) and the fear of infecting others (*p* = 0.001). Men rated discontinued and reduced face-to-face events as the biggest negative influencing factors on training conditions significantly more often (*p* = 0.035) and women discontinued and reduced in-house training (*p* = 0.031). Overall, men rated their personal well-being during the pandemic as bad to very bad in 64% (*n* = 34) and women in 26% (*n* = 45), but this did not reach statistical significance (*p* = 0.069; Supplementary Table 2).

Regarding the specialty, we distinguished surgical and non-surgical oncologic disciplines. There were significantly different answers to the question of whether working conditions changed during the pandemic because of more clinical work (surgeons *n* = 49, 32%; non-surgeons *n* = 134, 52%; *p* < 0.001), being transferred to a different working field (surgeons *n* = 42, 28%; non-surgeons *n* = 105, 41%; *p* = 0.006) and having less access to childcare (surgeons *n* = 13, 9%; non-surgeons *n* = 43, 17%; *p* = 0.018). Surgeons had alternatives to replace face-to-face education content significantly less often (*p* < 0.001) and rated discontinued and reduced elective interventions as the biggest negative influencing factors on training conditions significantly more often (*p* < 0.001) as well as being transferred to a different field of work (*p* = 0.001). Non-surgeons felt strongly burdened by excessive demands significantly more often (*p* = 0.043) and rated discontinued and reduced face-to-face events (*p* = 0.050) and in-house training (*p* = 0.003) as the biggest negative influencing factors on training conditions significantly more often.

Regarding the education level, participants with a higher degree (Dr. med. or PD) had significantly less changes in working conditions or scope of work (*p* = 0.027). Participants with a university degree felt strongly burdened by restricted communication with experienced colleagues significantly more often (*p* < 0.001).

## Discussion

In autumn 2022, the president of the German Cancer Society postulated that the coronavirus pandemic was not over, two and a half years after it began in Germany [29]. The pandemic had a significant impact on society, including the healthcare system and the way we had to learn to approach new challenges [29]. Many groups were affected and taken into account in publications and research, such as cancer patients, healthcare workers, apprentices and others [19, 21, 30, 31]. This led our group to raise the question of the current conditions in oncology training. We conducted an interdisciplinary survey to gain insight into the training situation of physicians in oncological disciplines during the pandemic. We explored questions such as the following: Did the pandemic alter training conditions? What factors influenced training the most? Did the quality of work and care for cancer patients suffer as a result? Ultimately, we aimed to draw conclusions that would enable us to improve oncological training in a crisis and beyond.

According to the survey results, the majority of respondents were female (62%). The largest proportion worked in radiation oncology (26%), were under 36 years old (58%) and were registrars (55%) at a university hospital (67%; Table 2 and Fig. 1).

In March 2020, a joint task force of the German Cancer Research Center (DKFZ), German Cancer Aid (DKH) and the German Cancer Society (DKG) was established to monitor oncological care capacity and identify bottlenecks [19]. The task force initiated a prospective panel study with the participation of 18 comprehensive cancer centres (CCC) in Germany, which together care for 15–20% of all new cancer cases each year. The feedback over more than 2 years (March 2020 to June 2022) shows limitations especially in follow-up care, psycho-oncology and tumour surgery compared to the “pre-corona” period [19].

According to evaluations of the population-based Bavarian Cancer Registry on the impact of the COVID-19 pandemic on the number of new cancer cases and treatments in Bavaria [32], the number of cancer treatments decreased statistically significantly by 4.0% for all cancer therapy types combined between January and September 2020 compared to 2019, and by as much as 6.1% for radiation treatments. Despite these detected limitations in cancer care, almost half of our participants stated that they had worked more clinically during the pandemic. According to our survey, this extra work was mainly due to more additional tasks (e.g., time-consuming hygiene concepts, carrying out COVID tests, issuing visiting permits), but also to a lack of staff during the pandemic. This is in line with the findings of the task force from DKFZ, DKH and DKG, which, at the time of our survey in spring 2022, identified staff shortages of 10–20% of regular staff capacity due to quarantine and self-isolation and limited capacity to admit additional patients [19]. According to our survey, the changed workload often resulted in less capacity for scientific work. Nearly 30% stated that they have or had no time for research during the pandemic. This suggests that the pandemic may have significantly impacted the ability of researchers to conduct research. There are several potential reasons for this, including the need to prioritize patient care, increased workload and reduced resources. These challenges may have made it difficult for researchers to dedicate time to research and may limit the personal development and career options of survey participants. However, it is unclear whether this trend will persist after the pandemic. As the situation improves and healthcare systems return to normalcy, researchers may have more time and resources to conduct research. Additionally, the pandemic may have highlighted the importance of research, leading to increased support for research and a renewed focus on its importance. To support researchers, additional funding, resources and support may be necessary. Alternative ways to conduct research may also need to be explored that are more resilient to future disruptions.

To reduce face-to-face contact, most meetings (team meetings, multidisciplinary meetings/tumour boards, in-house and external training and professional development events) took place virtually. Although tumour boards are primarily intended to guide treatment decisions for patient cases in a multidisciplinary meeting, we incorporated them within the educational framework in this survey because they also contribute to the trainee’s learning process. Attending tumour boards is highly advantageous for trainees despite their primary purpose not being educational. Compared to the time before the pandemic, more than a third of the respondents could attend these events considerably less often. Between 11 and 23% had no access at all to training sessions (Fig. 1). In particular, in-house events such as weekly training and journal club were partly cancelled without replacement (62% stated this), but could be transferred to alternative formats (65%). No access to internal training modalities almost doubled from 12% before to 23% during the pandemic (Fig. 1). The increase in the percentage of residents with no access to internal training modalities is a concerning trend that needs to be addressed. Potential reasons for this include changes in the training environment, a shift to alternative formats such as remote learning, limited resources due to staff and/or space shortage and lack of prioritization. Institutions should consider strategies such as reimagining the training environment, increasing access to technology, prioritizing training, and collaboration with other institutions to overcome these challenges and ensure that residents have access to necessary resources and training opportunities.

External events were almost totally transferred to alternative formats, which improved availability (42%). It is particularly noticeable here that this worsened professional exchange, personal contacts and networking (80% stated this), which, in turn, can limit personal development and career options.

Considering the large number of radiooncology participants (Table 2) and with regard to the comment fields, the webinars for exam preparation in radiation oncology developed due to the coronavirus pandemic (monthly since 02.12.2020) can be mentioned here as an external alternative training option (https://cutt.ly/D1YZMQa), as well as the numerous conversions of congresses or professional development events to hybrid formats.

In terms of hands-on training (interventional, surgical, diagnostic), access was limited and there was no replacement in more than half of the cases.

Fewer operations were performed overall, as an international (including Germany) prospective cohort study showed: of the more than 20,000 patients included who initially opted for curative surgery during the coronavirus pandemic, 10% did not undergo surgery after a median follow-up of 23 weeks. In 100% of these cases, a COVID-19-related reason was given for refusing surgery. One in seven affected persons in a region with complete lockdown did not receive planned surgery [30, 33].

Accordingly, alternative formats were also predominantly rated as poor in this category (66%). The loss of hands-on training in medical education, both during medical school and postgraduate education, is a critical issue that has been recognised by researchers and educators in the field. Practise recommendations have been provided by Dapper et al. [34]. This study sheds light on the current status of practical training in medical education and highlights the need for hands-on experiences to enhance clinical competence. Furthermore, Mäurer et al. [35] have introduced an interactive brachytherapy workshop as an example of a successful approach to addressing hands-on training in postgraduate medical education.

Virtual meetings were chosen as a good alternative to face-to-face meetings, especially in the areas of in-house and external professional development events, as well as for tumour boards (Fig. 2). During the pandemic, the curricula of medical universities also had to be converted to digital formats, which was efficiently possible in a recent single-centre analysis among students of radiation oncology and achieved good acceptance among the students [36]. Here, digital concepts enable university teaching or even deepen it. However, they prevent the direct interaction of students with real patients. A careful balance must be kept between digital and face-to-face training, also with regard to resident training.

In general, the participants had the necessary technical and spatial requirements and were not overburdened with them. It was easy for 41% of the respondents to train this way. Nevertheless, 33% felt that their training suffered using digital formats. Respondents were asked about possible changes regarding communication and feedback with their supervisors. Overall, the time for exchange and feedback was felt to be much less. The lack of feedback can make the training less specific. Evaluation on the status of training was mostly rated as bad (Fig. 3).

The importance of effective digital transfer in training and education currently plays a subordinate role in position and vision papers, but should be addressed in the future [37].

Regarding emotional well-being and psycho-social burden, the changed team atmosphere affected the respondents strongly in about half of the cases (Fig. 4a). The atmosphere, motivation and cohesion in the team suffered considerably during the pandemic. A bad mood in the team can have a negative impact on personal motivation, well-being and quality of work. More than one third of the colleagues additionally felt strongly burdened by the changed training conditions (Fig. 4a).

Other factors that caused considerable personal distress were the intensified hygiene procedures, including the wearing of masks, the (clinical) extra work, the initial uncertainty in dealing with the virus and the fear of infecting others with it (but not oneself), being absent due to illness and the changed personal environment, such as the lack of childcare facilities. Some of these reasons may have become less important as people familiarised themselves with the pandemic situation. Others, such as extra work and the lack of childcare facilities, were certainly dependent on the incidence figures and the associated quarantine/self-isolation and sickness levels [19].

There seemed to be an overall tendency for professional and personal strain to increase during the pandemic, but this only partially translated into a poorer sense of well-being in our respondents.

Almost half of the respondents rated their own motivation in the crisis situation as high, their own work satisfaction was better or worse in just over a third and just under a third stated poor personal well-being.

A worldwide survey of medical oncologists in the summer of 2020 showed even more significant limitations. Herein, 50% of respondents indicated that their general well-being had been severely impaired since the pandemic and 18% expected that they would not have recovered to baseline by the end of the year [38].

Although people might be concerned about the quality of medical care under pandemic conditions [21], in our survey, 64% (*n* = 174) of the participants rated their own quality of work as good and according to them, the pandemic-related changes had little influence on that (Fig. 6).

In our conducted subgroup analyses, gender was found to be a significant factor. Female participants felt a significant change in working conditions due to less access to childcare facilities during the pandemic compared to their male counterparts. Additionally, female participants faced challenges in elaborating cancelled education content on their own and from excessive demands at work. However, women were more likely to rate their work as good, whereas there was a trend toward men rating their personal well-being during the pandemic as worse. The findings related to gender align with the results of a scoping review [39] which explored women healthcare workers’ experiences during the COVID-19 pandemic and other crises, highlighting the disproportionate impact of the pandemic on women, including challenges related to childcare and increased workload.

Regarding specialty, our findings indicate that surgeons faced significantly more changes in working conditions due to increased clinical work, being transferred to different working fields and having fewer alternatives to replace face-to-face education content compared to non-surgeons. Non-surgeons felt significantly more burdened by excessive demands and identified the discontinuation of face-to-face events and in-house training as major negative factors affecting their training conditions.

In terms of education level, participants with higher degrees (such as Dr. med. or Priv.-Doz., PD) experienced significantly fewer changes in their working conditions or scope of work compared to those with a university degree. Conversely, participants with a university degree reported feeling significantly more burdened by restricted communication with experienced colleagues. This seems to be obvious in the sense that experienced colleges have to continue carrying out their duties, especially in the context of treating oncological patients. Early-career participants were probably more likely to work with COVID patients to support treatment of their rapidly increasing number during the beginning and ongoing pandemic and faced problems if they were no longer able to communicate with experienced colleagues.

The impact of the pandemic on healthcare worker education was also investigated in a recent systematic review and meta-analysis [40] that explored the global disruption, responses and lessons learned during the COVID-19 pandemic. The findings align with our results indicating significant changes in educational practices, including the discontinuation or reduction of face-to-face events and the need to find alternative methods for delivering education content.

Survey-based studies naturally have limitations, such as the subjectivity of the responses, the timing of the survey and other person-related reasons [41]. Our study may have a potential selection bias considering the high number of doctorates (Dr. med.) and habilitation/venia legendi (PD; together *n* = 329, 74%) as the highest educational qualification among the participants, which may indicate a high motivation for academic and scientific work.

All available options were used to disseminate the survey in order to reach the broadest possible spectrum of responses, knowing that a very heterogeneous spectrum of participants also means bias. It is not possible to ascertain with certainty whether individually named problems and grievances were caused by the coronavirus pandemic, aggravated by it or had already existed before but were now assessed differently.

Overall, more than one third of the participants stated that the coronavirus pandemic had a negative impact on oncology training (Fig. 6). The major negative factors were the discontinuation or reduction of face-to-face events, the discontinuation or reduction of in-house and external training options and the additional tasks that had to be carried out as a result of the pandemic. Reduced clinical time in the speciality area and the reduction of interventions or elective treatments were also rated as factors influencing training conditions negatively. The conducted subgroup analysis adds further granularity by considering the differential effects of gender, specialty and education level. These insights can inform the development of targeted interventions and support systems to address the specific needs of different groups within the healthcare workforce.

## Conclusion

In conclusion, the COVID-19 pandemic has had significant repercussions for oncology training and the healthcare system as a whole. The study’s findings indicate that training conditions have been altered. Access to face-to-face events and hands-on training as well as feedback and communication between supervisors and trainees was limited, posing concerns for the development of clinical competence. The emotional well-being and team atmosphere were negatively impacted, contributing to decreased motivation and cohesion. However, the majority of respondents rated their own quality of work still as good. To address the identified issues, a balanced approach between digital and face-to-face training should be adopted, ensuring access to necessary resources and technology. Achieving optimal quality of oncology training and medical care in times of crisis and beyond remains a challenge.

## Supplementary Information

Supplementary Tables 1 and 2
