# Supplementary material for: Impact of the COVID-19 pandemic on training conditions and education in oncologic disciplines: a survey-based analysis
Source: Strahlenther Onkol. 2023 Aug 4;199(9):806–19. doi: 10.1007/s00066-023-02121-6 (PMC10449661; doi:10.1007/s00066-023-02121-6)
Supplement: Supplementary file 1 — Supplementary Tables 1 and 2 [file 66_2023_2121_MOESM1_ESM.docx]

**Supplementary Table 1**: Differences in characteristics of the participants

| **Question** | **Group** | **Response n (%)** | **p value** |
| --- | --- | --- | --- |
| **working conditions during the pandemic** | | |  |
| no change of work scope or extent | |  |  |
|  | university degree | 23 (23%) | **0.027** |
|  | higher degree (MD, PD) | 109 (35%) |  |
| more clinical work | |  |  |
|  | surgical | 49 (32%) | **<0.001** |
|  | non-surgical | 134 (52%) |  |
| more research | |  |  |
|  | male | 31 (20%) | **0.008** |
|  | female | 27 (11%) |  |
|  | surgical | 31 (20%) | **0.006** |
|  | non-surgical | 27 (11%) |  |
|  | university degree | 7 (7%) | **0.020** |
|  | higher degree (MD, PD) | 51 (17%) |  |
| less elective interventions | |  |  |
|  | surgical | 59 (39%) | **<0.001** |
|  | non-surgical | 19 (7%) |  |
| transferred to different field (not original field of education) | | | |
|  | surgical | 42 (28%) | **0.006** |
|  | non-surgical | 105 (41%) |  |
| less access to childcare | |  |  |
|  | male | 14 (9%) | **0.032** |
|  | female | 42 (17%) |  |
|  | surgical | 13 (9%) | **0.018** |
|  | non-surgical | 43 (17%) |  |
| **in-house training** | |  |  |
| no accress before pandmic | |  |  |
|  | male | 10 (8%) | 0.058 |
|  | female | 31 (15%) |  |
|  | surgical | 24 (18%) | **0.068** |
|  | non-surgical | 17 (8%) |  |
| no access during pandemic | |  |  |
|  | male | 20 (16%) | **0.019** |
|  | female | 57 (27%) |  |
|  | surgical | 34 (26%) | 0.102 |
|  | non-surgical | 43 (21%) |  |
| no alternatives to replace face-to-face education content | | | |
|  | surgical | 78 (68%) | **<0.001** |
|  | non-surgical | 84 (46%) |  |
| **external training** | |  |  |
| no accress before pandmic | |  |  |
|  | university degree | 9 (11%) | 0.087 |
|  | higher degree (MD, PD) | 9 (4%) |  |
| no accress during pandmic | |  |  |
|  | university degree | 15 (19%) | **0.039** |
|  | higher degree (MD, PD) | 21 (9%) |  |
| **personal elaboration of cancelled education content** | | |  |
|  | male | 49 (44%) | **0.018** |
|  | female | 52 (28%) |  |
| **less feedback from supervisors** | |  |  |
|  | male | 55 (50%) | **0.009** |
|  | female | 68 (37%) |  |
| **personally rated quality of own work** | |  |  |
| good and very good | |  |  |
|  | male | 58 (57%) | **0.022** |
|  | female | 116 (67%) |  |
| **personal well-being** | |  |  |
| good and very good | |  |  |
|  | male | 38 (38%) | 0.069 |
|  | female | 63 (36%) |  |
|  | surgical | 43 (39%) | 0.286 |
|  | non-surgical | 58 (35%) |  |
| bad and very bad | |  |  |
|  | male | 34 (64%) | 0.069 |
|  | female | 45 (26%) |  |
|  | surgical | 25 (23%) | 0.286 |
|  | non-surgical | 44 (32%) |  |
| **pressure due to…** | |  |  |
| excessive demands | |  |  |
|  | male | 20 (20%) | **0.046** |
|  | female | 56 (31%) |  |
|  | surgical | 19 (17%) | **0.043** |
|  | non-surgical | 57 (33%) |  |
|  | university degree | 26 (39%) | 0.061 |
|  | higher degree (MD, PD) | 50 (23%) |  |
| less communication with experienced collegues | | |  |
|  | university degree | 35 (56%) | **<0.001** |
|  | higher degree (MD, PD) | 62 (30%) |  |
| fear to infect others | |  |  |
|  | male | 42 (41%) | **0.001** |
|  | female | 94 (52%) |  |
| **biggest negative influencing factors on training conditions** | | | |
| discontinuing/reducing face-to-face events | | |  |
|  | male | 44 (40%) | **0.035** |
|  | female | 60 (33%) |  |
|  | surgical | 33 (30%) | **0.050** |
|  | non-surgical | 71 (39%) |  |
| discontinuing/reducing in-house training | | |  |
|  | male | 39 (36%) | **0.031** |
|  | female | 79 (43%) |  |
|  | surgical | 36 (33%) | **0.003** |
|  | non-surgical | 82 (45%) |  |
| discontinuing/reducing elective interventions | | |  |
|  | surgical | 51 (46%) | **<0.001** |
|  | non-surgical | 22 (12%) |  |
| own abscence from work | |  |  |
|  | male | 18 (17%) | 0.078 |
|  | female | 52 (29%) |  |
| being transferred to different field of work | | |  |
|  | surgical | 36 (33%) | **0.001** |
|  | non-surgical | 35 (19%) |  |

**Supplementary Table 2**: Differences in answers of subgroups.
